# Just as the girl communicating with her mother using sign language: an electrocardiogram can convey a lot through its signals

**DOI:** 10.1093/ehjcr/ytae132

**Published:** 2024-03-15

**Authors:** Jayakrishna Niari, Nagesh Waghmare, Kalyan S Munde

**Affiliations:** Department of Cardiology, Grant Government Medical College and Sir JJ Group of Hospitals, JJ Marg, Mumbai Central, Mumbai, Maharashtra-400008, India; Department of Cardiology, Grant Government Medical College and Sir JJ Group of Hospitals, JJ Marg, Mumbai Central, Mumbai, Maharashtra-400008, India; Department of Cardiology, Grant Government Medical College and Sir JJ Group of Hospitals, JJ Marg, Mumbai Central, Mumbai, Maharashtra-400008, India

## Case

Upon entering the intensive care unit, I observed a young female patient communicating with her mother using sign language. Upon asking for a detailed medical history, I discovered that she is a 14-year-old who has been deaf and mute since childhood and has a history of syncopal episodes since the age of 7. Unfortunately, she was initially misdiagnosed with a seizure disorder at a local hospital and prescribed anti-epileptics. However, her syncopal episodes persisted, particularly during moments of anxiety or crying. Over the past year, she has experienced multiple episodes of syncope. Other than a normal cardiovascular system examination, her lab workup was unremarkable, except for the discovery of iron deficiency anaemia. A 12-lead electrocardiogram (ECG) was conducted, and the results are depicted in the figure.

**Figure:**
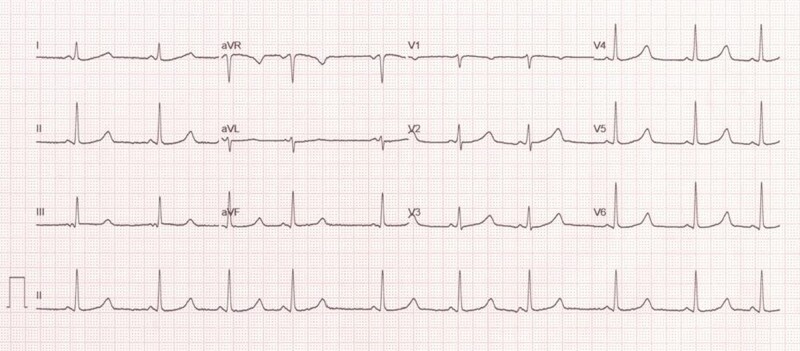


## Question 1

### What diagnosis is most closely represented in this ECG?

- Normal ECG
- Hypokalemia
- Jervell and Lange-Nielsen syndrome
- Romano–Ward syndrome
- Timothy syndrome

**Answer: C**

#### Discussion and explanation

The ECG reveals a QTc of >550 ms, with modified long QT syndrome (LQTS) score of 9, and a potassium level of 4.6 mEq/L. Jervell and Lange-Nielsen syndrome (JLNS) is identified as a rare autosomal recessive disorder characterized by a prolonged QTc interval, typically exceeding 480 ms, and congenital bilateral sensorineural hearing loss.^1,2^ The prolonged QTc interval in JLNS is linked to tachyarrhythmias, including ventricular tachycardia (VT), ventricular fibrillation, and episodes of torsade de pointes VT, which may result in syncope or sudden death. The classic presentation of JLNS involves a deaf child experiencing syncopal episodes triggered by exercise, stress, or fright.^1^

## Question 2

### What is the most common genetic abnormality present in this syndrome?

- SCN5A
- KCNE1
- KCNJ2
- KCNQ1
- CACNAC1

**Answer: D**

#### Discussion and explanation

The KCNQ1 mutation is associated with 90% of the cases, while the KCNE1 mutation is associated with only 10% of cases, and it is inherited as an autosomal recessive disorder.^3^ Our patient was found to have a KCNE1 mutation. Romano–Ward syndrome is associated with the same gene mutation but inherited as an autosomal dominant disorder with the absence of sensorineural deafness. Timothy syndrome is a multisystem disorder characterized by cardiac, facial, hand/foot, and neurodevelopmental features and is associated with a mutation in the CACNA1C gene. The KCNJ2 mutation is associated with Andersen–Tawil syndrome. SCN5A is associated with Brugada syndrome.^3^

## Question 3

### What are the appropriate treatment modalities for syncopal episodes in this patient?

- Beta-blockers alone
- ICD alone
- Beta-blockers, ICD
- Sympathetic denervation of the heart, ICD
- Mexiletene, ICD

**Answer: C**

#### Discussion and explanations

According to ESC guidelines, beta-blockers (ideally nadolol or propranolol) are recommended as the primary approach to reduce the risk of arrhythmic events.^2,4^ Implantable cardioverter defibrillator (ICD) therapy is recommended in patients with cardiac arrest and in patients who remain symptomatic despite on beta-blockers.^2^ As per the EHRA expert consensus, left cardiac sympathetic denervation is recommended for symptomatic patients despite beta-blockers, when ICD is contraindicated or declined, or for an ICD carrier who experiences multiple shocks or syncope due to ventricular arrhythmia while on beta-blockers.^2^ Mexiletine is indicated in LQT3 subtype.

Despite being treated with propranolol, our patient continues to have episodes of syncope, so an ICD was successfully placed using DDD mode (ST JUDE MEDICAL) (see Supplementary material online, *Material S1*). There have been no reported episodes of syncope since the implantation of the ICD over the past 3 years.

## Supplementary Material

ytae132_Supplementary_Data

## Data Availability

The data underlying this article are available in the article and in its online supplementary material.

## Supplementary material

**Consent**: The patient provided direct written consent for the inclusion of their information in this manuscript.

**Funding**: The development of this manuscript did not involve any external funding.
